# Oncolytic Virotherapy for Multiple Myeloma: Past, Present, and Future

**DOI:** 10.1155/2011/632948

**Published:** 2011-05-10

**Authors:** Chandini M. Thirukkumaran, Don G. Morris

**Affiliations:** Department of Oncology, Tom Baker Cancer Centre, 1331 29 Street NW, Calgary, AB, Canada T2N 4N1

## Abstract

Multiple myeloma (MM) is a B-cell malignancy that is currently felt to be incurable. Despite recently approved novel targeted treatments such as lenalidomide and bortezomib, most MM patients' relapse is emphasizing the need for effective and well-tolerated therapies for this deadly disease. The use of oncolytic viruses has garnered significant interest as cancer therapeutics in recent years, and are currently under intense clinical investigation. Both naturally occurring and engineered DNA and RNA viruses have been investigated preclinically as treatment modalities for several solid and hematological malignancies. Presently, only a genetically modified measles virus is in human clinical trials for MM. The information obtained from this and other future clinical trials will guide clinical application of oncolytic viruses as anticancer agents for MM. This paper provides a timely overview of the history of oncolytic viruses for the treatment of MM and future strategies for the optimization of viral therapy for this disease.

## 1. Introduction

Multiple myeloma (MM) is a clonal neoplasm of plasma cells derived from the B-lymphocyte lineage that is part of a spectrum of diseases ranging from monoclonal gammopathy of undetermined significance (MGUS) to plasma cell leukemia. It is the most common primary bone cancer and involves malignant plasma cells progressively infiltrating the bone marrow and producing a monoclonal immunoglobulin (Ig) (M-protein) [1]. Overt myeloma (advanced disease) is manifested by pathophysiological consequences such as osteolytic bone lesions, hypercalcemia, recurrent bacterial infections, anemia, and renal failure [2]. Over 70,000 people in North America are currently affected by MM with an annual incidence of greater than 15,000. Presently, MM accounts for 10% of hematological malignancies and represent 1-2% of all cancer-related deaths [3]. The disease remains incurable with current treatments with a median survival of 3–5 years [4, 5]. MM follows a relapsing course in the majority of patients, regardless of treatment regimen or initial response to treatment. Accordingly, it has become imperative to find novel, more effective treatment options for MM.

### 1.1. Currently Available Therapies for Multiple Myeloma

Disease management of MM has improved with the introduction of several new agents such as bortezomib (Velcade, a proteasome inhibitor), thalidomide, and the thalidomide analogue lenalidomide (Revlimid, immune modulator), and thus these drugs have now become current mainstays in MM treatment. These agents as monotherapies (bortezomib) or in combination (thalidomide or lenalidomide) with dexamethasone have yielded improved patient outcome, yet long-term tolerance and toxicities associated with these drugs are limitations [6].

Stem cell rescue following high-dose chemotherapy with autologous (ASCT) transplantation has historically become standard therapy for the subset of good-performance younger patients with MM. Yet minimal residual disease and/or contaminating tumour cells within the autograft leading to relapse is a concern with ASCT. 

### 1.2. A Historical Perspective of Oncolytic Viruses

The concept of virotherapy in the treatment of cancer dates back to the early 20th century and more recently with a report of virally mediated tumour regression involving a patient with cervical carcinoma that received an attenuated rabies vaccine [7]. Spontaneous remissions of heamatological malignancies such as Burkitt's lymphoma and Hodgkin's disease have also been observed subsequent to clinical infections with measles virus [8, 9]. Reports as early as the 1920s indicate that viral replication was responsible for consequent lysis of tumour in murine models [10]. Although anecdotal, these early observations provided the foundation to treat cancer patients with oncolytic viral therapy in the late 1940's; however, results were disappointing likely owing in part to the rapid viral clearance by the resultant induced immune response [11]. 

Despite these setbacks over the last 15 years, there has been a revival of interest in developing oncolytic viruses as potential cancer therapeutics. An increasing number of viruses have been shown to have oncolytic activity against both solid and haematological tumours *in vitro* and *in vivo*. The potential obstacles/limitations to a successful viable oncolytic therapeutic platform include: large-scale GMP viral production, toxicity, immunogenicity and optimization of the schedule/route of administration.

Naturally occurring or engineered oncolytic viruses have the theoretical therapeutic advantage over other cancer therapies in that they can specifically infect, propagate, and lyse cancer cells (including neighboring cancer cells) while sparing normal cells. Viral specificity depends on two major mechanisms to produce a productive viral infection and subsequent cell lysis/death: (i) receptor-mediated uptake, where cancer cells overexpress virus entry specific receptors, and (ii) Utilization of aberrant cellular oncogenic signaling pathways for virus replication. With the use of recombinant DNA technology, and our increasing understanding of microRNA functions, the capacity for creating novel “designer viruses” appears to be limitless and therefore very promising. Viruses genetically engineered to express suicidal genes, immune stimulatory products within the tumor and/or tumor-specific inflammatory responses, and limit replicative potential in normal cells are attractive candidates to be used as cancer therapeutics.

## 2. Viruses Used as Therapeutic Strategies for MM

Of the many viruses that are currently considered possible cancer therapeutics, four RNA viruses (measles virus, vesicular stomatitis virus, reovirus, coxsackievirus A21) and two DNA viruses (adenovirus and vaccinia virus) have been investigated as potential therapeutics for MM. These have been investigated preclinically as monotherapy, as combination therapy in conjunction with chemotherapy and/or radiation therapy and as purging agents during ASCT. 

Targeting multiple myeloma with virotherapy clinically was first attempted in the late 1980s, involving intravenous treatment of a Japanese male MM patient with an attenuated AS vaccinia strain that demonstrated significant reductions in his IgA monoclonal protein levels [12]. The first formal oncolytic viral MM clinical trial was conducted at the Mayo clinic with an attenuated oncolytic measles virus encoding thyroid sodium iodine symporter in order to improve sodium iodine uptake [13]. In addition, other naturally occurring viruses such as reovirus that is currently undergoing phase III clinical trial testing for solid tumour histologies are anticipated to undergo a phase I clinical trial for MM in the near future. 

### 2.1. Measles Virus (MV)

Measles virus is the most comprehensively studied oncolytic virus for MM and is the first to undergo phase I clinical trial investigation for this disease. It is a negative-strand RNA virus of the genus *Morbillivirus* that causes the infectious measles syndrome. Its genome consists of 6 genes that encode 8 proteins: the nucleocapsid (N), phospho (P), matrix (M), fusion (F), hemagglutinin (H), and large (L) proteins in addition to C and V accessory proteins. The virus enters cells through the interaction of H-glycoprotein with the CD46 receptor that is overexpressed in cancer cells including MM [14, 15] and the signaling lymphocyte-activation molecules (SLAM) that are found in B- and T-lymphocytes [16]. After receptor recognition by the H-protein, the conformational changes that take place in the F-protein lead to viral entry and cell fusion. Cytopathic effects of MV are mediated by massive cell fusion due to virus receptor recognition and the formation of syncytia (large mononuclear cell aggregates) [17]. The MV-Edm (Edmonston vaccine strain) is a replicating measles strain that was isolated from an 11-year-old pediatric patient and hitherto named after him. This virus has been attenuated after serial tissue culture passage and has been administered as a vaccine for over 50 years. Reversal of this strain to a virulent form has never been reported, and thus it has been exploited as cancer therapeutics based on its longstanding safety profile. 

#### 2.1.1. Preclinical Studies with MV

The success of MV-Edm has been in part linked to the mutations of the H-glycoprotein that leads to improved interactions with the CD46 receptor [18], and it is of interest that over expression of CD46 in cancer cells has been correlated with enhanced cell death [19]. 

The earliest *in vitro* and *in vivo* work with MV-Edm and MM was carried out by Peng et al. in 2001 [20]. This study demonstrated effective lysis of MM cell lines *in vitro *as well as patient MM tumor utilizing a GFP-tagged MV-Edm, with no adverse effects on normal blood lymphocytes. MM tumours implanted in a SCID/NOD murine xenograft model also showed complete tumour regression following intratumoural MV treatment and significant tumour response with intravenous virus treatment. 

A major drawback of standard viral vectors is the poor delivery efficiency, especially in a clinical setting. Several strategies have been adopted with MV for better delivery and to produce enhanced bystander effects that will facilitate the killing of tumour cells that are shielded from virus. The MV-Edm derivatives utilizing human carcinoembryonic antigen (CEA; MV-CEA) or human sodium iodide symporter (NIS; MV-NIS) have successfully been utilized to produce significant local bystander effects through cell-cell fusion and syncytia formation [21–25]. 

In addition to enhancing tumour targeting strategies, these engineered derivatives provide a means of tracking the *in vivo* spread of the virus and virus gene expression/kinetics over time. These parameters provide insight for tailoring viral delivery doses and frequencies of repeated cycles which could lead to a personalized approach of treatment. While both MV-NIS and MV-CEA allow expedient real-time monitoring of viral gene expression, MV-NIS has an additional advantage in that it carries a membrane ion channel (NIS) that can concentrate radioisotopes within cells thereby causing localization and the spread of the virus. This unique advantage of isotope trapping by NIS facilitates the non-invasive detection of the spread of the virus by *γ* camera, PET, or SPECT/CT by using radioisotope tracers such as ^123^I, ^124^I, and ^99M^Tc [26]. 

The oncolytic potential of MV-NIS against MM was investigated by Dingli et al. 2004 [22]. *In vitro* MM cell lines and *ex vivo* MM tumour specimens showed marked sensitivity, and the therapeutic efficacy of MV-NIS against MM xenografts was noteworthy as previously MV-NIS resistant MM1 tumours showed complete regression of tumour with combination therapy with ^123^I.

#### 2.1.2. Clinical Studies with MV

The preclinical efficacy and the safety data generated for MV-NIS have translated these findings to a phase I clinical trial for recurrent or refractory MM that is ongoing at present [13]. This trial includes the intravenous administration of MV-NIS with or without cyclophosphamide. In addition to being a chemotherapeutic drug, cyclophosphamide is an immune suppressor, and thus prolonged viral dissemination and replication within these immune-suppressed patients are expected as previously seen in animal models [27]. A 2-step protocol for the phase I clinical trial was adapted where the maximum tolerated dose is to be evaluated in the first step where patients will be given intravenous injections of MV-NIS ranging from 10^6^ to 10^9^ TCID-50. The second step will commence once the MTD is reached where patients in groups of 3 will be pretreated with cyclophosphamide two days prior to MV-NIS injection. Pre and posttherapy hematological and biochemical parameters as well as antimeasles immunity will be determined in the patients. In addition to measurements of MV-NIS levels in blood, urine, and gargle samples, patients would undergo serial imaging of virus biodistribution post-^123^I administration [13]. CD46 expression of patient myeloma cells isolated from bone marrow will be correlated with virus infectivity in order to validate previous laboratory *in vitro* studies. Interestingly, unpublished observations as reported by Msaouel et al. [26] indicate the uptake of ^123^I in a localized MM tumour of one patient as revealed by a SPECT/CT8 scanning 8 days after treatment. These encouraging results would hopefully lead to advance phase II/III clinical trials in the future.

### 2.2. Reovirus

Reovirus is a ubiquitous, nonenveloped double-stranded RNA virus with minimal pathogenicity in humans [28]. Although, in newborn and severely immunocompromised (SCID) mice, reovirus type 3 may cause encephalitis, myocarditis and death [29–32], immunocompetent animals including humans have never exhibited any of these toxicities [33]. Since reovirus is a common environmental virus, the vast majority of humans have neutralizing antibodies to this virus by the age of 8 [34]. Reovirus is internalized into cells via the ubiquitous sialic acid receptor [35] and/or the junction adhesion molecule (JAM) [36]. It uses a strategy of cell infection and lysis through exploitation of an already activated Ras/oncogenic signalling pathway in tumor cells [35]. Thus, reovirus specifically targets tumor cells for its replication and spares normal cells. 

#### 2.2.1. Preclinical Studies with Reovirus

The underlying mechanism(s) behind the preferential cytotoxicity of reovirus towards transformed cells has only recently been described and appears to be at the level of intracellular signalling and not at cell surface attachment [35]. When reovirus resistant murine NIH 3T3 cells are transformed with oncogenes such as *v-erb*B_2_, *sos* and *ras*, reovirus susceptibility is conferred [35, 37]. Reovirus likely exploits an activated Ras/oncogenic signalling pathway, taking advantage of the inhibition of the double-stranded RNA activated protein kinase (PKR) found in these cells [35]. Recent data has implicated the Ras/RalGEF/p38 pathway in an NIH 3T3 model system of reovirus oncolysis [38]. 

 The oncolytic ability of reovirus against several neoplasms including breast, prostate, colorectal, brain, ovarian, and hematological malignancies such as non-Hodgkin's lymphoma, chronic lymphocytic leukemia (CLL), and MM has been shown by our group under *in vitro, in vivo,* and/or *ex vivo* conditions [39–44]. 

As in other tumor types, activating mutations in the *ras* gene family are frequent in MM [45, 46]. The incidence of activating M-*ras *and K-*ras* mutations appears to be common in MM and varies between 10 and 40% at presentation, but may be as high as 70% at the time of relapse, indicating not only a possible role in tumor progression but also potential for reovirus sensitivity. Our laboratory exploited these features of MM and investigated the potential of reovirus as biological therapeutics against MM. Our initial studies showed RPMI 8226 MM cell line and an *ex vivo* patient tumour to be sensitive to reovirus [44]. Expanding on these findings, we have tested 8 MM cell lines and 7 *ex vivo* patient tumour samples *in vitro* and found 7 of 8 human MM cell lines and 5 of 7 *ex vivo* tumor specimens exposed to reovirus to be exquisitely sensitive [47, manuscript submitted]. In addition, we have shown that the potent antitumour efficacy of reovirus is predominantly manifested through apoptotic cell death [48]. These results indicate the potential use of this virus as attractive therapeutic for MM. 

Most MM patients have symptomatic disease at diagnosis, and autologous hematopoietic progenitor stem cell transplantation (ASCT) is applicable for more than 50% of patients with MM [49]. Stem cell rescue after high-dose ablative therapy has proven to be an effective and useful treatment modality for a variety of hematologic malignancies including MM as well as a few solid tumours [50–56]. Because of its low treatment-related mortality rate (<3%) and the absence of the need for a suitable donor (allotransplantation), autologous transplantation has gained widespread application, and globally the number of autologous blood and marrow transplants now surpasses the number of allotransplants [55, 57, 58]. 

Although ASCT following high-dose myeloablative chemotherapy is considered standard therapy for many multiple myeloma (MM) patients, relapse post-ASCT still presents a major challenge in disease management. Gene-marking studies indicate that occult clonogenic tumour cells within the autograft may be a partial contributor to relapse [59].

Since MM currently remains the second most common indication following lymphoma for autotransplantation [60], we explored the possibility of using reovirus as a purging agent for MM. In significant contrast to its sensitivity towards MM tumour, we have shown that reovirus does not harm hematopoietic stem cells or their colony-forming abilities *in vitro *or* in vivo*. Furthermore, RPMI 8226 cells admixed with human apheresis product (AP) cells showed complete purging when treated with reovirus. To examine the potential use of this strategy under clinical conditions, we recently utilized a murine model system that recapitulated the human course of MM and demonstrated that reovirus purged autografts do not abrogate human hematopoietic stem cell repopulation *in vivo*. Further, we have shown that reovirus purging leads to complete eradication of disease, prevents relapse, and leads to significant survival improvements in comparison to controls [47, manuscript submitted]. 

#### 2.2.2. Clinical Studies with Reovirus

Upwards of 16 Reovirus (REOLYSIN) phase I/II clinical trials in several cancers have shown moderate efficacy, especially in combination with radiotherapy and histology relevant cytotoxic chemotherapy [61–66], and phase III trials are presently ongoing for non-small cell lung carcinoma and head and neck cancers [66]. Reovirus's extensive preclinical efficacy, replication competency, and low toxicity profile in humans have placed it as attractive anticancer therapeutics for further clinical testing for hematological malignancies. We anticipate that the preclinical data generated for MM will lead to a clinical trial of reovirus in MM and possibly a reovirus purging trial in the near future. 

### 2.3. Coxsackievirus A21 (CVA21)

CVA21 is a nonenveloped, positive-sense single-stranded virus that belongs to the *Picornaviridae* family. Although it is known to cause respiratory tract infections and myositis in humans [67, 68], its oncolytic potential has been proven in several cancer cell lines including MM [69, 70]. Intracellular adhesion molecule-1 (ICAM-1) and decay-accelerating factor are 2 receptors that are necessary for CVA21 infection and cell lysis, and both these are reported to be upregulated in cancer cells including MM in comparison to normal cells [69, 71]. CVA21-mediated cell death is manifested through interruption of various cellular processes such as disruption of cellular protein synthesis, abrogation of transport of cellular glycoproteins, proteolytic digestion of transcription factors, and promotion of apoptosis [72]. 

#### 2.3.1. Preclinical Studies with CVA21

Work conducted by Au et al. [69] showed that RPMI 8226, U266, and NCI-H929 cell lines were exquisitely sensitive to CVA21 producing 100–1000 fold increases in viral progeny at 24h after infection compared to controls. Normal peripheral blood cells in contrast were resistant to virus infection. Similarly, patient bone marrow biopsies exposing to CVA21 lead to purging of CD138+ plasma cells up to 98.7% with minimal effects on progenitor function [69]. Since CVA 21 can cause severe myositis in suckling [73] and immunocompromised mice [70], Kelly et al. [70] utilized a microRNA- (miRNA-) based approach to decrease virus pathogenicity. In this study virus tropism was modulated with the expression of tissue- (muscle-) specific miRNA within the engineered CVA21 thereby destabilizing viral replication in a tissue-specific manner. SCID mice bearing subcutaneous Kas 6/1 MM tumours injected with miRT-CVA21 showed complete tumor regression and sustained viremia but could not replicate in cells containing complementary miRNAs and therefore did not cause myositis. This study shows that naturally occurring and differentially expressing miRNAs can be exploited to modulate viral replication cycles, thus providing a new paradigm of virotherapeutics.

#### 2.3.2. Clinical Studies with CVA21

To date, no clinical investigations have been initiated with CVA21 and MM. However, a phase I clinical study is currently underway for patients bearing melanoma, breast and prostate tumor that express cellular receptor ICAM-1 with or without DAF expression [74]. The preclinical data reported to date suggest that CVA21 has the potential to purge MM during ASCT or be used as a systemic virotherapy agent for MM and warrants further investigation.

### 2.4. Vesicular Stomatitis Virus (VSV)

Vesicular stomatitis virus (VSV) is a small negative-strand enveloped RNA virus that belongs to the *Rhabdoviridae* family. With a wide host range, its ability to cause vesicular lesions in farm animals is common [75]; however, the incidence of human infections is rare [76] and usually benign [77]. Naturally occurring VSV is highly sensitive to interferon (IFN) and exploits inherent IFN dysregulated pathways in tumour cells for its replication and eventual tumour destruction [78]. 

#### 2.4.1. Preclinical Studies with VSV

Lichty et al. [79] have shown several leukemic cell lines and multiple myeloma *ex vivo* patient specimens to be exquisitely sensitive to VSV variants AV1, AV2 and heat resistant (HR) VSV [79]. In addition, this group also demonstrated that leukemic cell lines could be purged successfully with these VSV variants with minimal effect on colony-forming ability of hematopoietic stem cells suggesting potential use of these VSV mutants as purging agents [79]. To date, *in vivo* testing of the *ex vivo* purging efficacy of these VSV variants has not been undertaken.

Due to its small size, VSV is amenable to genetic manipulation, and recently VSV∆51 has been engineered to express the human sodium iodide symporter (hNIS) for combined imaging and radiotherapy of MM [80]. VSVΔ51-hNIS generated by Goel et al. [80] was oncolytic to MM cell lines, as well as primary patient tumours, and produced very high titers in MM cells under *in vitro* conditions. VSVΔ51-hNIS administered to bg/nd/xid mice bearing subcutaneous myeloma tumors demonstrated significant tumour regression, and high intratumoral virus replication was noted [80]. 

Utilizing a syngeneic 5TGM1 murine model of MM, Goel et al. [80] further demonstrated the *in vivo* oncolytic ability of VSVΔ51-hNIS where subcutaneous or orthotopic tumours treated with VSVΔ51hNIS in combination of ^131^I showed marked reduction of tumour and improved rates of survival of mice.

#### 2.4.2. Clinical Studies with VSV

Since MM is a radiosensitive tumour, *in vivo* work conducted with VSV∆51hNIS suggests the potential usage of this virus in combination therapy for clinical use in the future for MM.

### 2.5. Vaccinia Virus (VV)

Vaccinia virus belongs to the family *poxviridae *and is a close relative of the smallpox virus [81]. It is a double stranded DNA virus with a large genome that is (of 190 KB) amenable to genetic manipulation. Due to its strong immunogenic nature (that results in high T-cell responses and circulating antibodies), VV has been utilized as a vaccine that is instrumental in eradicating smallpox [82]. Various VV strains have further been exploited in immunotherapy of cancer and infectious diseases and as cancer therapeutics itself [82, 83]. 

#### 2.5.1. Preclinical Studies with Vaccinia Virus (VV)

The thymidine kinase (*TK*) gene of VV serves as a site of DNA insertion, and the first logical oncolytic VV was developed by McCart et al. [84] which is highly attenuated. In this double mutant VV, the *TK *and vaccinia growth factor (VGF) genes have been deleted and the gene for enhanced green fluorescent protein (EGFP) has been inserted at the TK locus resulting in a double-deleted GFP construct (vvDD-GFP). This attenuated virus's tumour selectivity, safety profile, and oncolytic effects have been evaluated in MM recently [85]. MM cell lines and *ex vivo* patient tumour exposed to vvDD-GFP showed sensitivity, whereas minimal viral infectivity was seen in normal peripheral blood mononuclear cells [85]. Systemic VV treatment of mice bearing My5 subcutaneous xenografts or RPMI 82226 disseminated tumour showed significant reduction in tumour and improved survival over the controls suggesting the potential use of this virus as a clinical agent in the future.

#### 2.5.2. Clinical Studies with Vaccinia Virus

The first clinical trial involving MM and VV virotherapy was a case study conducted with a 67-year-old Japanese male patient with IgA MM in the late 1980s [12]. Intravenous injections of vaccinia strain AS administered to this patient resulted in marked reductions in his IgA levels from 1,309 mg/dl in the early stages of treatment to 432 mg/dl on the 96th day of the regimen. No adverse effects were noted. 

Since then, other clinical trials of VV mutants such as JX-594 have been conducted in patients with refractory primary or metastatic liver cancer with indications of efficacy [83]. JX-594 is a targeted, thymidine kinase(-) vaccinia virus expressing human granulocyte-macrophage colony-stimulating factor (hGM-CSF) and is designed to selectively replicate in and destroy cancer cells with cell-cycle abnormalities and epidermal growth factor receptor- (EGFR-) Ras aberrant signaling pathways. GM-CSF expression by JX-594 stimulates shutdown of tumour vasculature and antitumoral immunity in addition to direct oncolysis. The encouraging clinical results seen with JX-594 will hopefully lead to future clinical trials in hematological malignancies including MM.

### 2.6. Adenovirus

Adenoviruses (Ad) are non-enveloped double-stranded DNA viruses, and wild-type Ad may cause mild clinical infections of the upper respiratory tract; however, may cause significant morbidity and mortality in immune-compromised patients. The majority of studies involving adenovirus as an oncolytic agent have utilized attenuated nonreplicative adenoviral vectors that have been engineered either to deliver a prodrug activating enzyme such as thymidine kinase (*TK*) or express wild-type p53 selectively in tumour cells (reviewed in [86]). The tumour cell selectivity of Ad is explained by the presence of the coxsackievirus and Ad receptor (CAR receptor) on tumour cells in association with the expression of *α*_v_ *β*_5_ or *α*_v_ *β*_3_ integrins required for internalization of these virions into the tumour cell [87]. Modification of the viral attachment fibre knob [88] is an additional strategy that has been utilized for selective entry of Ad into tumour cells.

#### 2.6.1. Preclinical Studies with Adenovirus and MM

 Teoh et al. [89] showed that Ad vectors carrying the thymidine kinase gene (*TK*) under the DF3 promoter could transduce OCI-My5 and RPMI8226 MM cell lines efficiently. Treatment of these cells in the presence of normal hematopoietic progenitor (HPC) cells could lead to a >6 log purging of tumour cells leaving the HPC cells unharmed. Similarly, Ad-mediated delivery of p53 to MM cell lines or patient tumour resulted in substantial apoptosis if the cells were p53 mutant with low expression of bcl-2 [90]. In contrast, HPC cells or normal lymphocytes were not permissive to these Ad vectors [90]. Replicating Ad1337 with E1A and E1B deletions has also been shown to cause cytotoxicity to MM cell lines but not normal B cells [91]. More recently, Fernandez et al. [92] examined growth inhibition of MM cells potentiated by a conditionally replicating adenovirus carrying a CD40 ligand transgene (AdEHCD40L). Their work demonstrated that MM cell lines were susceptible to AdEHCD40L-mediated apoptosis. RPMI 8226 xenografts in a SCID murine model were reduced by 50% by AdEHCD40L treatment, whereas treatment with the vector alone showed only a 28% reduction in tumour. Since Ad5 serotype has been approved for human use in solid tumours, Senac et al. [93] investigated the potential use of Ad5 for MM. Their work demonstrated that Ad5 could infect and kill the majority of myeloma cell lines and *ex vivo *patient tumour as evidenced by reporter gene expression, viral DNA expression, viral titre, and cell death assays. When MM patient specimens were exposed to different adenovirus species, many showed the capability to kill MM tumour of patient origin and thus suggested significant therapeutic potential. 

Although the therapeutic advantages of adenoviruses as an oncolytic agent for MM under clinical conditions are yet to be investigated, the efficacy and safety profile of many adenoviral vectors during phase I/II clinical trials demonstrated for solid tumours is of some concern, and whether or not this virus could be exploited for MM therapy in the future is unknown.  Table 1 depicts a comparison of oncolytic viruses that have been investigated for MM.

## 3. Optimization of MM Treatment with Oncolytic Virotherapy

In the clinical setting, oncolytic viral therapy needs to be ideally administered intravenously (IV) to MM patients due to its hematological route of disease progression. The main obstacles that prevent successful delivery of IV-injected virus to tumour sites are recognition and irreversible clearance of viral antigens by the immune system, nonspecific clearance of virus by the liver and spleen, and minimal extravasation of virus from the blood vessels to the targeted tumour site. Various strategies have been developed by researchers to overcome these barriers, and these are discussed in detail below. 

### 3.1. Immune Modulation

A major drawback of virotherapy under clinical settings is that the majority of patients either have baseline or generate neutralizing antibodies against the therapeutic virus of interest. Overcoming this immune responses would greatly enhance the therapeutic efficacy of virotherapy. Although malignancy-reported immunosuppression is common in MM patients [94], it is imperative to develop strategies to overcome host immune viral responses if virotherapy is to be optimized. To this end, cyclophosphamide has been shown to be a suitable immunosuppresant in animal models as well as in early clinical trials with MV, herpes virus as well as reovirus [27, 95–99]. Preclinical data of MV-NIS in squirrel monkeys has shown prolonged viral gene expression with cyclophosphamide administration to animals [27]. Similar administration of cyclophosphamide has shown to dampen the innate immune responses and increase the therapeutic efficacy of herpes virus with prolonged viral gene expression and enhance proliferation in tumor [95]. Clinical studies with reovirus in combination with cyclophosphamide are presently being conducted in UK for advanced malignancies of pancreatic, lung, and ovarian cancer http://www.oncolyticsbiotech.com/clinical.html. The primary objective of this study is to determine the minimum effective immunomodulatory dose (MED) of cyclophosphamide to obtain successful immune modulation. The secondary objective is the safety profile and obtaining any evidence of antitumour activity.

### 3.2. Cell Carrier Based Methods

Cell-based viral carriers are another approach that has been developed to circumvent immune clearance of therapeutic viruses. Mesenchymal progenitor cells, monocytes, and T cells have demonstrated the ability to shield virus from immune invasion and traffic them to tumour sites leading to enhanced oncolysis [100–102]. 

Ong et al. [100] evaluated T cells as carriers for systemic measles virotherapy for MM in the presence of antiviral antibodies. This study demonstrated that virus-infected T cells expressing measles H/F fusogenic envelope glycoproteins could efficiently transfer MV infection by heterofusion with MM cells *in vitro*. These T cells were more efficient in delivering virus to MM tumour sites than naked viruses in mice that were passively immunized with low levels of antimeasles antibodies illustrating a similar strategy that may be feasible with MM patients harbouring low immunity. 

Another strategy that has been experimented is that the virus of choice could also multiply within the carrier cell, disperse and infect adjacent tumour cells. A major advantage of MM cells to be used for such a strategy is that they express chemokines that are necessary to home to bone marrow thereby facilitating nondestructive trafficking of virus to sites of tumour target. 

Since MM cell lines express CXCR4 and home to bone marrow and tumour cells of similar histological origin are reported to bind to one another [102–105], Munguia et al. [102] exploited these features and recently utilized MM cell lines themselves as viral delivery vehicles to tumour sites. In this study, irradiated murine 5TGM1 MM cells were still susceptible to VSV-GFP infection and were able to produce progeny virus at levels similar to nonirradiated cells. Utilizing an orthotopic human myeloma (KAS/61 cell) model they showed that VSV-GFP-infected myeloma cells administered systemically could travel to sites of myeloma tumor growth supporting the feasibility of this strategy for future use in the clinic.

## 4. Conclusions/Future Directions

 During the past decade, the oncolytic viral platform has expanded vastly resulting in the testing of many new naturally occurring and engineered viruses as possible therapeutics for MM. The present paper describes encouraging investigation into oncolytic viruses for therapeutic agents in the treatment of MM. In an era of personalized medicine, MM represents an attractive target for viral therapy over many solid tumours due to being an easily accessible tumour where the dissemination of disease is prevalent in bone marrow and blood. Therefore, the suitability of a viral treatment as monotherapy, as combination therapy (with chemotherapy and/or radiation therapy), or as a purging agent could be evaluated *ex vivo* before systemic treatment of patients. Although the optimal strategy utilizing oncolytic viruses for the treatment of MM is unknown, it is anticipated that the use of those viruses will not be used as monotherapy. The combination of oncolytic viruses plus relevant cytotoxic or targeted drug may well prove to be synergistic. Further, one might envision that oncolytic virus treatment could be used post- allo/-auto transplant once hematopoietic recovery has been achieved. 

It appears from the advances made to date that the future of oncolytic virotherapy lies in combination therapies (versus monotherapy) for this malignancy, and a rationale for testing novel drugs for MM in combination with oncolytic viruses is justified. A major obstacle that hinders virotherapy is the host immune system that has evolved over the millennia in to overcome infection. The many strategies that researchers have undertaken to circumvent this problem such as immune modulation, shielding the virus in cell carrier, and so forth. have provided insight into overcoming these barriers. Many of the viruses mentioned in the aforementioned discussion are presently under phase II/III testing for solid tumors, and phase I clinical trials are ongoing with measles virus for MM. It is expected that the outcomes of these studies will facilitate to further testing of these viruses for MM in a clinical setting and be approved as cancer therapeutics in the near future.

## Figures and Tables

**Table 1:** Genetic composition, advantages, and disadvantages of oncolytic viruses presently evaluated for the treatment of MM.

	Measles virus	VSV	Reovirus	CVA21	Adenovirus	Vaccinia virus
Genetic composition	ssRNA	ssRNA	dsRNA	ssRNA	dsDNA	dsDNA

Ability to genetically manipulate	Easy	Moderate	Very difficult	Moderate	Easy	Easy

Titres achievable at clinical grade	>10^9^ PFU/ml	>10^9^ PFU/ml	>10^9^ PFU/ml	>10^10^ PFU/ml	>10^12^ PFU/ml	>10^9^ PFU/ml

Ease of production	Easy	Difficult	Easy	?	Easy	Easy

Preclinical	References	References	References	References	References	References
*in vitro *	[20, 22]	[79, 80]	[44, 47]	[69, 70]	[89–93]	[85]
*ex vivo *	[20, 22]	[80]	[44, 47]	[69]	[92, 93]	[85]
*purging *		[79]	[44, 47]	[69]	[89]	

Preclinical						
*in vivo *	[20, 22, 23]	[80]	[47]	[70]	[92, 93]	[85]

Clinical studies						
Multiple myeloma	Phase I (13)	N/A	Under discussion	N/A	N/A	Case study (12)
Other histologies	Recurrent glioblastoma multiforme, recurrent ovarian cancer (26)	N/A	Phase III for solid tumours (66)	Phase I- for melanoma, breast, prostate (74)	Phase I/II for several solid tumours and melanoma (106)	Phase I for primary or metastatic liver cancer

Strategies for delivery of virus for MM	Intravenous	Intravenous	Intravenous	Intravenous	Intravenous	Intravenous

VSV: vesicular stomatitis virus; CVA2: coxsackie virus A21; N/A: not available.
